# Adherence to Psoriasis Remote Monitoring and Its Associations With Demographic, Clinical, and Health Care Use Characteristics: Multicenter Observational Study

**DOI:** 10.2196/89706

**Published:** 2026-08-13

**Authors:** Maarja Lember, Priit Kruus, Katrin Kaarna, Tanel Traks, Laura Prett, Liisi Raam, Oliver Taul, Liis Ilves, Kaisa Viljar, Pille Konno, Secil Matasova, Peeter Ross, Külli Kingo

**Affiliations:** 1 Tallinn University of Technology Tallinn, Harjumaa Estonia; 2 Dermtest OÜ Tallinn, Harjumaa Estonia; 3 University of Tartu Tartu, Tartu Estonia; 4 Tartu University Hospital Tartu, Tartu Estonia; 5 OÜ Linnamõisa Perearstikeskus Tallinn, Harjumaa Estonia; 6 Confido Medical Centre Tallinn, Harjumaa Estonia

**Keywords:** psoriasis, remote monitoring, electronic patient-reported outcome, ePROM, adherence, eHealth, insurance database

## Abstract

**Background:**

Psoriasis is a lifelong, relapsing, quick-to-flare skin disease that requires personalized, continuous management. Due to its unpredictable nature, there is an increasing interest in using electronic patient-reported outcome measures (ePROMs) for remote monitoring of the condition. However, adherence to ePROMs and predictors of adherence have not been sufficiently explored among patients with psoriasis.

**Objective:**

This study aimed to assess the adherence to ePROMs and the factors associated with adherence among patients with psoriasis.

**Methods:**

We conducted an observational study that spanned multiple centers to measure adherence to ePROMs among 55 patients managed in primary care and 55 patients managed in specialized care over 12 months. We analyzed the data together with longitudinal health care data recorded in Estonia’s countrywide health insurance database—a unique dataset previously unavailable to researchers studying remote monitoring. While continuing their routine clinical care, patients were periodically prompted to complete ePROMs. Adherence to remote monitoring was evaluated in relation to a range of demographic, clinical, and health care use factors using ordinal logistic regression.

**Results:**

A total of 102 patients (male: n=49, 48%; female: n=53, 52%) were included in the adherence calculation, including 46 (45.1%) patients in the high-adherence group, 22 (21.6%) in the medium-adherence group, and 34 (33.3%) in the low-adherence group. The total mean adherence ratio was 63.4% (SD 30.8%). The mean adherence ratio was 60.9% (SD 32.8%) among patients in primary care and 66.0% (SD 28.9%) among patients in specialized care. In multivariable ordinal logistic regression, older age was associated with higher adherence (*P*=.03). No significant associations were observed for the other investigated factors.

**Conclusions:**

This study adds to existing research on the use of ePROMs in chronic diseases by demonstrating the feasibility of using ePROMs for remote monitoring of chronic skin diseases across different levels of care. The findings provide insights into factors associated with adherence, which may inform the development of tailored approaches to improve the effectiveness of remote monitoring in psoriasis. Importantly, adherence remained strong among older adults, supporting broader implementation of ePROM-based remote care.

## Introduction

Remote monitoring with routine patient-reported outcome measures (PROMs) is an effective strategy for capturing vital disease information for efficient patient-physician communication [1]. Remote monitoring makes timely tracking of symptom progression possible [2] and raises physicians’ awareness of patients’ perceived health status [3], resulting in improved patient-centered care [4].

Remote monitoring with electronic PROMs (ePROMs) is also being considered for implementation in the care process for psoriasis [5-7], a chronic, remitting, and relapsing immune-mediated skin disease affecting more than 60 million people of all ages worldwide [8,9]. Psoriasis is associated with a range of comorbidities of varying severity, including psoriatic arthritis [10-15]. Psychological distress in patients is caused both by the visible presence of lesions on the skin and by the subjective experience of lesion-related pain and itching [16]. The severity of psoriasis fluctuates throughout a person’s lifetime, and health care needs vary depending on disease severity [17]. The disease requires personalized ongoing monitoring and management to increase the duration of remission, improve quality of life [9,17], and reduce the risk of comorbidities [18-20]. Monitoring psoriasis with ePROMs shows promise in equipping physicians with better information [21].

The chronic and variable progression of psoriasis poses significant challenges to developing effective remote monitoring programs for its management [22-24]. eHealth solutions are potentially susceptible to low adherence [25], and the rate of patient correspondence “with agreed recommendations from a health care provider,” in the words of a World Health Organization report [26], can be even lower in psoriasis monitoring [27]. Reaching sufficiently high adherence, which Donkin et al [28] defined as “the degree to which the user followed the program as it was designed,” assumes that the program needs proper operational design and fits the health care context [29].

Understanding the possible factors that can hinder or support adherence is therefore important to ensure successful program design and sufficient adherence. Adherence to eHealth solutions is increasingly investigated, as inconsistent adherence limits the potential benefits of these solutions and could negatively impact treatment efficacy [25]. However, there are limited studies about which quantitative factors affect adherence to remote monitoring with ePROMs [30], specifically in chronic inflammatory skin disorders.

Previous research on adherence to remote monitoring systems or the predictors of high adherence among patients with psoriasis is limited. This study design is unique compared to existing remote monitoring programs in terms of its complexity (2 intervention groups, one at the primary care level and the other at the specialized care level, across 9 locations) and the range of factors investigated. In this paper, we focused on remote psoriasis management with ePROMs to study adherence and adherence factors based on countrywide health insurance fund claims data. The data from all patient insurance claims across Estonia were linked with the adherence results of patients in the program. The recruitment period for remote monitoring was from January 2022 to June 2022, followed by 12 months of monitoring for each patient. Adherence to remote monitoring with ePROMs among patients with psoriasis treated in either primary care or a dermatology outpatient clinic over 12 months was analyzed, and possible predictive factors for adherence were identified.

## Methods

### Study Design

An observational study used multisite monitoring data collected from January 2022 to June 2023. The publicly reimbursed monitoring was conducted across 7 primary care centers and 1 dermatology outpatient department at Tartu University Hospital, all in Estonia. While patients continued with routine clinical care, they were requested to complete validated ePROMs commonly used in psoriasis management, including the Psoriasis Symptoms and Signs Diary (PSSD) [21], the Dermatology Life Quality Index (DLQI) [31], and the Early Arthritis for Psoriatic Patients (EARP) screening questionnaire [18]. Patients also had the opportunity to add photos to the questionnaires.

The web application could be used with any browser, either on a computer or a mobile device (iOS and Android). Patients were notified through email when a new ePROM became available for answering and were provided with a link to the web application. On the first day of every month, the PSSD was requested; on the 15th day of every month, the DLQI was requested; and the EARP was requested on days 90 and 270. The web application transmitted patient-generated data to a secure online platform. Physicians were notified and could then access the results, review them, and use the information as part of standard care provision.

A previous publication reported results from the same cohort and evaluated the effectiveness of remote psoriasis monitoring across primary and specialist care levels, demonstrating that specialist-led care was associated with significantly greater improvements in quality of life [32]. In contrast, the present study focuses on adherence to remote monitoring and aims to identify factors associated with successful ePROM completion.

### Participants and Recruitment Procedure

In total, 110 patients were initially registered—55 (50%) from the dermatology outpatient department and 55 (50%) from 7 primary care centers. Eligible individuals were identified during an outpatient or primary care visit with their regular physician responsible for managing their psoriasis. Inclusion criteria were as follows: (1) male and female outpatients aged 18 to 75 years, (2) plaque psoriasis diagnosed by a physician, (3) access to a computer or smartphone with internet access and a digital camera, and (4) an understanding of the study time commitment and provision of informed consent. Exclusion criteria were as follows: (1) a psoriasis subtype other than plaque psoriasis, (2) a history of alcohol or drug abuse within the 6 months prior to the study [33], (3) inability to comply with study requirements, and (4) other unspecified reasons that made the patient unsuitable for inclusion based on the discretion of the investigator.

For onboarding, the following activities were performed for each patient: completion of the physician-assessed Psoriasis Area and Severity Index (PASI) [34] questionnaire by the physician and initiation of the patient’s remote monitoring protocol on the application platform. During the initial face-to-face onboarding visit, the physician was required to ensure that the patient could complete an ePROM via the web application. This was accomplished by sending an ePROM prompt to the patient’s email and instructing them to complete it.

### Determining Adherence Ratio

The adherence ratio was presented as a percentage and was determined by calculating the ratio of reported electronic patient-reported outcomes (ePROs) to the total number of ePROs that could have been reported [25]. Adherence was operationalized as “the number of necessary ePROMs completed and submitted within 30 days of receiving notification” [29]. This completion window was selected to evaluate overall engagement with remote monitoring over the 12-month period rather than strict compliance with scheduled dates. Considering the nature of the questionnaires, delays within this time frame were not expected to substantially affect the reported outcomes.

PSSD, DLQI, and EARP results were collected from the eHealth company database. Data were cleaned to eliminate any duplicate or incomplete entries for PSSD, DLQI, and EARP responses. The response dates of the ePROMs were assessed to ensure that responses were submitted within a 30-day period of receiving notification. ePROs completed during face-to-face onboarding sessions were excluded from the adherence calculations as these did not accurately reflect patient-initiated adherence behavior. Adherence was assessed for each participant and summarized across the sample. Adherence was categorized into 3 categories: low (<50%), medium (50%-75%), and high (>75%).

### Predictors of Adherence and Statistical Analysis

The characteristics of the study cohort are presented in Table 1. The investigated demographic data comprised sex and age. Psoriasis-specific factors comprised disease duration, treatment status, and baseline PASI scores. Investigated health care factors included the number of psoriasis-related comorbidities, patients’ care level (primary and specialized care), and the total number of unique health care services used during the study period (primary care contact visits or primary care remote consultations and specialist contact visits or specialist remote consultations). The Estonian Health Insurance Fund (EHIF) data (ie, health care service type and service dates) were cross-checked with the patients’ individual study dates. All comorbidity data pertaining to the year 2022 were comprehensively incorporated into the investigated dataset to minimize the risk of overlooking any diagnoses. Treatment data used in this study were collected from prescriptions fulfilled by patients during their individual study dates and the preceding 3-month period. The psoriasis duration for each patient was calculated from the first recorded psoriasis diagnosis code in the EHIF database.

Factors potentially associated with adherence were evaluated using multivariable ordinal logistic regression. Multicollinearity was assessed using variance inflation factors (VIFs) calculated from a linear regression model including all predictors. The proportional odds assumption was evaluated for each variable using a parallel lines test. PASI data were available for a subset of participants (n=86, 84.3%). Therefore, PASI was not included in the primary regression model to preserve statistical power. As a sensitivity analysis, the ordinal logistic regression was repeated among participants with available PASI data, with PASI included as an additional predictor. The stability of the regression coefficients and odds ratios (ORs) was compared between the primary and sensitivity models to ensure the robustness of the conclusions. All statistical analyses were conducted using Jamovi (version 2.6.44) with the GAMLj module for advanced generalized linear modeling diagnostics. A *P*<.05 was considered statistically significant.

**Table 1:** Characteristics of the study population (N=102).

Characteristics			Values
**Sex, n (%)**			
	Male	49 (48)	
	Female	53 (52)	
Age (years), mean (SD)			44.4 (10.4)
Psoriasis duration (years), mean (SD)			10.8 (6.3)
**Treatment, n (%)**			
	Topical only	64 (62.7)	
	Systemic or biological	7 (6.9)	
	Systemic and topical	13 (12.7)	
	No treatment	18 (17.6)	
Baseline Psoriasis Area and Severity Index score, mean (SD)			4.7 (6.3)^a^
**Comorbidities, n (%)**			
	Hypertension	14 (13.7)	
	Cardiovascular disease	10 (9.8)	
	Dyslipidemia	12 (11.8)	
	Psoriatic arthritis	12 (11.8)	
	Mood disorders (depression and anxiety)	9 (8.8)	
	Obesity	8 (7.8)	
	Type 2 diabetes	2 (2)	
	Autoimmune thyroiditis	3 (2.9)	
**Care level, n (%)**			
	Primary care	51 (50)	
	Specialized care	51 (50)	
Primary care clinic consultations, mean (SD)			2.5 (3.1)
Primary care remote consultations, mean (SD)			4.8 (3.5)
Specialist clinic consultations, mean (SD)			1.7 (1.6)
Specialist remote consultations, mean (SD)			0.2 (0.5)

^a^Psoriasis Area and Severity Index data were available for 86 participants.

### Ethical Considerations

The project was approved by the research ethics committee of the University of Tartu, Estonia (350/T-14). Eligible patients who understood the study time commitment were asked to provide informed consent. To protect privacy and confidentiality, the study used only anonymized data that had undergone a process of anonymization by authorized personnel involved in the study. Data were initially pseudonymized during queries of the respective databases, after which they were merged into a single database and then anonymized by assigning a new unique identifier to each patient. No incentives or compensation were offered to the patients.

## Results

Among the 110 patients initially registered for the study, 8 (7.3%) did not complete any ePROM questionnaires. Consequently, the adherence analysis included 102 (92.7%) participants who initiated the use of the remote monitoring program and contributed adherence data. The remaining cohort had an almost equal distribution of male (n=49, 48%) and female (n=53, 52%) participants. On the basis of the PASI scores at the start of the study, 68 (66.7%) patients had no to mild psoriasis (PASI <7) [35]. Prevalent comorbidities found in our sample population were hypertension and other cardiovascular diseases, dyslipidemia, psoriatic arthritis, mood disorders, obesity, type 2 diabetes, and autoimmune thyroiditis (Table 1). Patient characteristics did not differ significantly between primary care and specialist care, except for the higher proportion of patients in specialized care who received topical, systemic, or biological therapy, as shown in Table S1 in Multimedia Appendix 1.

The number of patients in each adherence group was as follows: 46 (45.1%) patients in the high-adherence group, 22 (21.6%) in the medium-adherence group, and 34 (33.3%) in the low-adherence group. The overall mean adherence ratio was 63.4% (SD 30.8%). The mean adherence ratio was 60.9% (SD 32.8%) among patients in primary care and 66.0% (SD 28.9%) among patients in specialized care. There was a gradual decrease in the mean adherence ratio during the 12-month monitoring period, from 74.0% (SD 27.2%) in the first quarter to 62.8% (SD 39.3%) in the final quarter. Average adherence to the monthly ePROMs was 65.3% (SD 31.7%) for the DLQI and 64.2% (SD 32.7%) for the PSSD, whereas average adherence to the less frequently administered EARP was 47.5% (SD 39.4%). In the primary ordinal logistic regression analysis (N=102), increasing age was independently associated with higher ePROM adherence (OR 1.05, 95% CI 1.01-1.09; *P*=.03; Table 2). No other predictors reached statistical significance, although a greater number of primary care remote consultations and receipt of specialist care showed a trend toward significance (*P*=.09 and *P*=.09, respectively). Multicollinearity among the included predictors was negligible, with all VIF values remaining <2. Parallel lines tests indicated that the proportional odds assumption was generally satisfied, with only primary care clinic consultations showing evidence of deviation (*P*=.02). PASI data were available for 86 (84.3%) participants and therefore were not included in the primary model. A secondary sensitivity analysis was restricted to participants with available PASI data and included PASI as an additional predictor (Table 2). The baseline PASI score was not associated with ePROM adherence (*P*=.81). Importantly, the inclusion of PASI did not meaningfully alter the model coefficients, and age remained a statistically significant predictor with an identical effect size (OR 1.05, 95% CI 1.00-1.10; *P*=.04). This finding supports the robustness of the primary findings and demonstrates their independence from baseline disease severity.

**Table 2:** Multivariable ordinal logistic regression analysis of factors associated with adherence to electronic patient-reported outcome measures (ePROMs).

Adherence factors	Primary model (N=102)			Sensitivity model (N=86)	
	Odds ratio (95% CI)	*P* value	Odds ratio (95% CI)		*P* value
Sex (female vs male^a^)	1.15 (0.52-2.57)	.73	0.99 (0.42-2.34)		.99
Age (years)	1.05 (1.01-1.09)	.03^b^	1.05 (1.00-1.10)		.04^b^
Psoriasis duration (years)	0.98 (0.92-1.04)	.48	0.98 (0.91-1.04)		.48
Treatment status (treatment vs no treatment^a^)	0.65 (0.21-2.00)	.46	0.89 (0.25-3.17)		.85
Baseline Psoriasis Area and Severity Index score	—^c^	—	0.99 (0.92-1.07)		.81
Number of comorbidities	0.97 (0.67-1.44)	.89	1.03 (0.67-1.58)		.91
Care level (specialized vs primary^a^)	2.39 (0.88-6.74)	.09	1.99 (0.71-5.70)		.19
Number of primary care clinic consultations	1.04 (0.90-1.23)	.59	0.98 (0.80-1.18)		.79
Number of primary care remote consultations	1.13 (0.99-1.29)	.09	1.04 (0.90-1.19)		.63
Number of specialist clinic consultations	1.01 (0.78-1.32)	.97	0.97 (0.72-1.31)		.84
Number of specialist remote consultations	0.60 (0.24-1.37)	.25	0.53 (0.19-1.30)		.20

^a^Reference category.

^b^Significant difference (*P*<.05).

^c^Not estimated because the variable was not included in the model.

## Discussion

### Principal Results

The overall mean adherence ratio of 63.4% (SD 30.8%; N=102) is comparable to adherence rates of 49% to 96% reported in other studies that used repetitive ePROMs in noncommunicable disease management [36-42], although ePROMs have not been studied in the context of psoriasis. These findings suggest that patients with psoriasis can successfully engage in regular remote monitoring with ePROMs in both primary and specialized care settings. Average adherence decreased from 74.0% (SD 27.2%) during the first quarter to 62.8% (SD 39.3%) during the final quarter, suggesting reduced engagement over time. Lower overall completion rates were observed for the EARP, which was requested twice during the monitoring period, than for the monthly DLQI and PSSD. This may indicate that participants perceived it as less relevant if they did not experience musculoskeletal symptoms related to psoriatic arthritis.

Most of the factors examined in the countrywide insurance claims registry were not associated with adherence, which is broadly consistent with prior studies that evaluated similar factors when analyzing adherence to PROs [36-42]. However, the results showed an association between increasing age and adherence (*P*=.03).

Previous studies have reported mixed findings regarding the relationship between older age and higher adherence compared to younger individuals. Our finding is therefore not entirely consistent with previous literature, although a systematic review of 5 studies on telemonitoring adherence identified 1 (by Colls et al [41]) showing “that patients over 65 years old had higher adherence compared to patients <45 years old” [30]. The other studies covered in the mentioned systematic review did not show age as a predictor of adherence. Possibly relevant, another study detected an age bias in email-based PROM recruitment, reporting that older patients were more likely to respond [43]. Although age was not significantly associated with adherence in that sample, the older participants who joined may represent a more motivated and responsive subgroup, indicating survivor or adopter bias.

Other investigated factors (sex, disease duration, baseline psoriasis severity, treatment status, number of comorbidities, care level [primary or specialized], and number of health care consultations [across primary and specialist care, both clinic based and remote]) were not associated with adherence, which is consistent with existing literature on adherence to ePROM-based monitoring of chronic diseases, although some discrepancies exist. For example, in patients with rheumatoid arthritis, older age [41,42] and lower symptom severity [41] have been related to better adherence, whereas higher symptom severity has also been associated with better adherence when coupled with chronic pain [40]. Similarly, primary care management was identified as an adherence facilitator in patients with heart failure [37], while our study did not identify any significant differences in adherence between care levels. A publication using the same dataset demonstrated clear differences in outcomes between primary and specialist care groups [32], while the current paper did not find significant differences in patient adherence between the 2 care levels.

Previous adherence studies have often had an overrepresentation of female participants [38-41], but this study had an equal sex composition. Despite the finding that female patients had a higher average adherence ratio than male patients (mean 64.1%, SD 33.3% vs mean 62.7%, SD 28.6%), sex was not a statistically significant predictor of adherence, consistent with prior studies [37-41]. The findings imply that future research should concentrate on comprehensive data collection incorporating additional categories of factors that can significantly influence adherence, such as patient-level demographics, including marital status, educational level, employment status, and income [44]. Moreover, contextualizing nonadherence through interviews and personalizing remote monitoring with psychosocial or educational strategies could be useful for developing more effective, tailored interventions for chronic conditions such as psoriasis.

### Limitations

The adherence factors investigated were limited to those accessible from the EHIF database, providing a unique set of potential predictors gathered at a national level that lacks some factors often examined in adherence studies. Additionally, potential attrition and volunteer biases due to purposive sampling, data cleaning, and nonrandomization were not investigated. However, we acknowledge the possibility that such biases might exist, as volunteering patients may have had different attitudes toward completing the ePROMs than nonvolunteering patients. Furthermore, the requirement for smartphone or computer access may have selected more digitally capable patients, including older adults. In addition, the study population was largely homogeneous, which meant that the generally underexplored sociocultural and racial determinants of health could not be assessed. Therefore, this may limit the generalizability of our findings to more diverse populations. Literature indicates that eHealth solutions incorporating psychosocial or educational components have demonstrated increased quality of life [7,45,46], and these elements were not included as part of our intervention. Thus, a key limitation is the lack of data on education, income, marital status, digital literacy, work status, motivation, satisfaction, and technology access, all of which may influence adherence and should be examined in future research. Another limitation is the absence of baseline PASI data for 15.7% (16/102) of participants. Consequently, disease severity could only be evaluated in a sensitivity analysis conducted on a reduced sample, although inclusion of PASI did not materially alter the findings. The regression analyses included a relatively large number of potential predictors in relation to the sample size. Although this may have reduced the precision of individual coefficient estimates and increased the risk of overparameterization, the variables were selected to examine their associations with adherence rather than to develop a predictive model. The observed association between age and adherence remained consistent in the sensitivity analysis, including PASI, supporting the robustness of the primary finding.

### Conclusions

This study contributes to the unique niche of improving the understanding of remote monitoring with ePROMs in patients with psoriasis, providing insights for selecting factors and designing quality interventions. Although some studies have evaluated remote monitoring as a long-term, sustainable management option for different chronic conditions, there is a general lack of research on the penetration, acceptability, and longitudinal sustainability of remote monitoring in psoriasis. In this study, 2 equal-sized intervention groups with very similar characteristics across primary and specialist care levels showed similar adherence. Of the 11 factors investigated, only 1 factor was found to be associated with adherence. Thus, the study builds on existing research while highlighting the massive need for future studies to expand the scope of possible adherence predictors, including psychosocial and socioeconomic factors.

Remote monitoring is an underused method of disease management that could potentially lead to improved clinical outcomes. However, the effect of the delivery methods for ePROMs, as well as the sociological factors that may play into the disease perception, is understudied. Multifaceted research that investigates adherence in conjunction with individual disease perception, education, and clinical outcomes is necessary to realize the full potential of remote monitoring.

## Appendix

Multimedia Appendix 1 Adherence predictive factors among primary care and specialized care patients.

## Abbreviations

DLQI: Dermatology Life Quality Index
EARP: Early Arthritis for Psoriatic Patients
EHIF: Estonian Health Insurance Fund
ePRO: electronic patient-reported outcome
ePROM: electronic patient-reported outcome measure
OR: odds ratio
PASI: Psoriasis Area and Severity Index
PROM: patient-reported outcome measure
PSSD: Psoriasis Symptoms and Signs Diary
VIF: variance inflation factor
