# Supplementary material for: Adherence to Psoriasis Remote Monitoring and Its Associations With Demographic, Clinical, and Health Care Use Characteristics: Multicenter Observational Study
Source: JMIR Form Res. 2026 Aug 13;10:e89706. doi: 10.2196/89706 (PMC13522812; doi:10.2196/89706)
Supplement: Supplementary file 1 [file formative_v10i1e89706_app1.docx]

**Supplementary**

**Table S1.** Adherence predictive factors among primary care and specialised care patients.

| **Characteristic** | **Primary care (n=51), No. (%) or mean (SD)** | **Specialised care (n=51), No. (%) or mean (SD)** | **Test** | ***P*-value** |
| --- | --- | --- | --- | --- |
| Sex (female) | 31 (60.8%) | 22 (43.1%) | χ² | 0.074 |
| Age | 43.7 (9.2) | 45.1 (11.5) | Student’s T | 0.496 |
| Duration | 11.1 (5.9) | 10.6 (6.7) | Mann–Whitney U | 0.775 |
| Treatment (at least one modality) | 36 (70.6%) | 48 (94.1%) | χ² | 0.002* |
| Baseline PASI^a^ score | 4.9 (7.0) | 4.5 (5.7) | Mann–Whitney U | 0.598^b^ |
| Number of comorbidities | 0.7 (1.0) | 0.7 (1.2) | Mann–Whitney U | 0.900 |
| Hypertension | 7 (13.7%) | 7 (13.7%) | Fisher’s exact | 1.000 |
| Cardiovascular disease | 4 (7.8%) | 6 (11.8%) | Fisher’s exact | 0.741 |
| Dyslipidaemia | 3 (5.9%) | 9 (17.6%) | Fisher’s exact | 0.122 |
| Psoriatic arthritis | 8 (15.7%) | 4 (7.8%) | Fisher’s exact | 0.357 |
| Mood disorders (depression, anxiety) | 5 (9.8%) | 4 (7.8%) | Fisher’s exact | 1.000 |
| Obesity | 5 (9.8%) | 3 (5.9%) | Fisher’s exact | 0.715 |
| Type 2 diabetes | 0 (0.0%) | 2 (3.9%) | Fisher’s exact | 0.495 |
| Autoimmune thyroiditis | 2 (3.9%) | 1 (2.0%) | Fisher’s exact | 1.000 |

^a^ PASI - Psoriasis Area Severity Index

^b^ PASI data were available for 86 participants.

* Significant difference (*P*<.05)
